# Safety and efficacy of applying sufficient analgesia combined with a minimal sedation program as an early antihypertensive treatment for spontaneous intracerebral hemorrhage: a randomized controlled trial

**DOI:** 10.1186/s13063-018-2943-6

**Published:** 2018-11-06

**Authors:** Rui Dong, Fen Li, Ying Xu, Pingyan Chen, Marc Maegele, Hong Yang, Wenjin Chen

**Affiliations:** 1 Department of Intensive Care Unit, The Third Affiliated Hospital of Southern Medical University, No.183 West Zhongshan Ave, Tianhe District, Guangzhou, 510630 Guangdong China; 2 Department of Biostatistics, School of Public Health, Southern Medical University, 1838 North Guangzhou Avenue, Guangzhou, 510515 China; 3 Institute for Research in Operative Medicine (IFOM), Witten/Herdecke University (Campus Cologne-Merheim), Ostmerheimerstr. 200, 51109 Cologne, Germany; 4 Department of Neurosurgery, Xuanwu Hospital, Capital Medical University, 45 Changchun Street, Xicheng District, Beijing, 100053 China

**Keywords:** Intracerebral hemorrhage, Early antihypertensive treatment, Sufficient analgesia, Minimal sedation, Clinical trial

## Abstract

**Background:**

Spontaneous intracerebral hemorrhage (ICH) is a serious threat to human health. Although early blood pressure (BP) elevation is closely associated with a poor prognosis, the optimal antihypertensive regimen for acute-phase ICH remains controversial. In ICH, pain, sleep deprivation, and stress are usually the main causes of dramatic BP increases. While traditional antihypertensive treatment resolves the increased BP, it does not address the root cause of the disease. Remifentanil relieves pain and, when combined with dexmedetomidine’s antisympathetic action, can restore elevated BP to normal levels. Here, we seek to validate the efficacy and safety of applying sufficient analgesia in combination with a minimal sedation program versus antihypertensive drug therapy for the early and rapid stabilization of BP in ICH patients.

**Methods/design:**

We are conducting a multicenter, prospective, randomized controlled, single-blinded, superiority clinical trial across 15 hospitals. We will enroll 354 subjects in mainland China, and all subjects will be randomized into experimental and control groups in which they will be given remifentanil combined with dexmedetomidine or antihypertensive drugs (urapidil, nicardipine, and labetalol). The primary endpoint will be the systolic BP control rate within 1 h of treatment initiation, and the efficacy and safety of the antihypertensive regimens will be compared between the two groups. Secondary endpoints include the incidence rate of early hemorrhage growth, neurological function, duration of intensive care unit (ICU) stay, and staff satisfaction with the treatment process.

**Discussion:**

We hypothesize that applying sufficient analgesia in combination with minimal sedation will act as an effective and safe antihypertensive strategy in ICH and that this treatment strategy could, therefore, be widely used as an ICH acute-phase therapy.

**Trial registration:**

ClinicalTrials.gov, ID: NCT03207100. Registered on 22 July 2017.

**Electronic supplementary material:**

The online version of this article (10.1186/s13063-018-2943-6) contains supplementary material, which is available to authorized users.

## Background

Spontaneous intracerebral hemorrhage (ICH) is a hemorrhage occurring in the brain parenchyma that was caused by the non-traumatic spontaneous rupture of a cerebral artery, arteriole, vein or capillary in an adult. ICH is classified as primary or secondary ICH based on its etiology. Primary ICH accounts for 80–85% of all cases of spontaneous ICH and mainly includes hypertensive ICH (50–70%) [1], amyloid angiopathy-associated ICH (20–30%), and ICH of unknown cause (10%). Secondary ICH includes ICH caused by a venous malformation, aneurysm, arteriovenous fistula, cavernous malformation, hematological disorder or coagulopathy, hemorrhagic cerebral infarction, venous sinus thrombosis or adverse drug reaction or Moyamoya disease [2]. ICH accounts for 25–55% of brain stroke cases in Asian countries and 10–15% of such cases in European and American countries [3]. Increased blood pressure (BP) has already been confirmed as a risk factor for ICH development and a poor prognosis [4, 5]. A systematic review showed that a 10-mmHg increase in systolic BP caused a 42% increase in the risk of ICH [5]. Approximately 90% of ICH patients experience an immediate increase in BP after disease onset [6–8]. BP elevation in the acute phase of ICH is associated with a poor prognosis, is caused by mechanisms of action, such as a local increase in the initial hemorrhage or an early hematoma expansion at hemorrhagic sites [9–14], and is associated with an increased risk of early recurrent hemorrhage, serious cerebral edema [15, 16], and recurrent stroke [17]. In an observational study of 1701 patients, patients with a mean arterial pressure of 134 mmHg on admission were more likely to have fatal outcomes, while those with a mean arterial pressure of 120 mmHg on admission were more likely to survive [18]. The current American Heart Association guidelines suggested that inducing a rapid decrease in BP to 140 mmHg is safe in ICH patients with no obvious antihypertensive contraindications [19–21]. However, the results of several large randomized controlled trials (RCTs) completed in recent years have presented significantly different findings [22–25]. A meta-analysis that included 4360 subjects enrolled in five RCTs showed that intensive acute BP lowering (to a target BP of 140 mmHg) was safe but did not seem to provide an increased clinical benefit in terms of functional outcomes [26]. These results were substantially different from those presented in previous studies, and differences in inclusion criteria (i.e., enrolled patients exhibited small ICH volumes and a small number of warfarin-associated hematomas) and complications in addition to differences in early BP control rates and variability in BP increases may represent major causes of the inconsistencies observed among these studies [20–25]. Because the wide range of BP-lowering medications used among these studies have different modes of administration, it was not possible to identify an optimal antihypertensive therapy for rapid and stable BP lowering in ICH [26].

Some studies have shown that the stress response, pain, and intracranial pressure (ICP) increases are factors that cause acute BP increases in ICH patients [19, 27]. The primary principles underlying treatment for acute BP increases in ICH are to keep the patient quiet, restore BP to a normal level, decrease BP variability, and lower the chance of recurrent hemorrhage [23, 28, 29]. Combining analgesia and sedation is a critical component of this strategy for which there is global consensus [19, 30]. Remifentanil is a fentanyl μ-type opioid-receptor agonist with strong and fast-acting analgesic effects that does not induce ICP elevation [31], and dexmedetomidine is an α_2_-adrenergic agonist that inhibits sympathetic activity and reduces norepinephrine release [31]. Remifentanil and dexmedetomidine are mainly used as anesthetic and analgesia/sedation medications in critical patients. ICH is one of the most common critical diseases, and analgesia/sedation has been shown to control pain and anxiety and to provide a neuroprotective effect in the acute phase of ICH [31]. Therefore, our research group developed a treatment strategy in which sufficient analgesia is applied in combination with a minimal sedation program as an effective and safe early antihypertensive treatment. We hypothesize that applying sufficient analgesia in combination with a minimal sedation program will involve the use of remifentanil for pain relief and dexmedetomidine for antisympathetic activity to restore elevated BP to normal levels in patients with spontaneous ICH, and we further hypothesize that this strategy will be more effective than conventional symptomatic antihypertensive treatment for controlling BP.

## Methods/design

### Study design

The present study is a multicenter, prospective, randomized controlled, single-blinded (participant), superiority clinical trial. Subjects in the experimental group will be treated with remifentanil combined with dexmedetomidine to reduce systolic BP to 110–140 mmHg. In the positive drug-controlled group, routine antihypertensive medications (e.g., urapidil, nicardipine, and labetalol) will be used to lower BP to the target level. This clinical study is compliant with the Declaration of Helsinki and the relevant regulations for clinical research in China. This clinical trial protocol has been approved by the Institutional Ethics Committee (IEC) of The Third Affiliated Hospital of Southern Medical University. The trial is registered under NCT 03207100.

#### Patient recruitment and consent procedure

Patients will be recruited from 15 clinical centers in mainland China to participate in the trial. The participating hospitals and centers of the trial are listed in Table 1. Since the subjects of interest of this study are primarily acute ICH patients with an unforeseeable disease onset, the placement of recruitment advertisements in the trial centers is, therefore, not appropriate for this study. As an alternative, we provided training to all executors at the 15 trial centers so that the executors are able to provide information about the study and conduct subject recruitment when they encounter emergency ICH patients. All study participants will be provided an informed consent form (ICF) that briefly describes the study so they can decide whether to participate in the study. Each participant will be explicitly informed that participation in the study is voluntary, that they may withdraw from the study at any time and that withdrawal of consent will not affect their subsequent medical assistance and treatment. Before participation, all enrolled participants will be asked to provide a signed ICF. The process of informed consent will be conducted by local investigators. A clinical research inspector will be delegated by the Ethics Committee of The Third Affiliated Hospital of Southern Medical University to monitor all processes of informed consent performed during this study. Inspections will be conducted once per every four subjects enrolled, and all inspection results will be submitted to the Ethics Committee. Of note, because ICH can impair the consciousness of the subject in subjects with impaired consciousness or cognitive impairment, informed consent can be acquired from the subject’s immediate family members or a legal guardian.

**Table 1:** Participating hospitals and centers of the trial

Participating hospitals and centers	
Xuanwu Hospital Capital Medical University	
The Third Affiliated Hospital of Southern Medical University	
Qilu Hospital of Shandong University	
First Affiliated Hospital of Kuming Medical University	
The First Affiliated Hospital of Xinjiang Medical University	
Xinqiao Hospital of Army Medical University	
Henan Provincial People’s Hospital	
Xiangya Hospital Central South University	
The Second Hospital University of South China	
Nanfang Hospital	
The First Affiliated Hospital of Jinan University	
The Second People’s Hospital of Shenzhen	
The People’s Hospital of Guangxi Zhuang Autonomous Region	
The Second Affiliated Hospital of Zhejiang University School of Medicine	
The First Hospital of Lanzhou University	

#### Primary and secondary objectives

The primary objective of this study was to validate the efficacy and safety of applying a sufficient amount of analgesia in combination with a minimal sedation program as an early rapid antihypertensive treatment in ICH patients. Secondary objectives are to evaluate the efficacy of this treatment program for reducing early hematoma expansion and improving the short-term prognosis of ICH patients, to evaluate its superiority in improving work satisfaction among critical unit healthcare workers, and to evaluate its effect on deterioration in neurological function and a reduction in adverse reactions in ICH patients.

### Inclusion and exclusion criteria

#### Inclusion criteria

1. Acute stroke syndrome due to spontaneous ICH, which is defined as the sudden occurrence of bleeding into the parenchyma of the brain that may extend into the ventricles or subarachnoid space as confirmed by a clinical history and computed tomography (CT) scan [27]
2. Systolic BP ≥ 150 mmHg at least twice (based on brachial artery pressure measured in the upper arm in two measurements conducted ≥ 2 min apart)
3. Age > 18 years
4. Feasible for emergency antihypertensive treatment and real-time BP monitoring
5. Symptom onset within 24 h (if the time of onset is unknown, the last known time is selected); and
6. Intensive care unit (ICU) or stroke unit admission within 24 h

#### Exclusion criteria

1. Subjects with contraindications for emergency-intensified antihypertensive treatment (such as severe carotid, vertebral or cerebral artery stenosis, known Moyamoya disease or multiple arteritis, and severe aortic stenosis or severe kidney failure)
2. Intracranial hemorrhage secondary to intracranial tumor, recent trauma, cerebral infarction and thrombolytic therapy
3. History of ischemic stroke within 30 days before disease onset
4. A clinical or imaging examination revealing findings associated with high mortality within the next 24 h (e.g., Glasgow Coma Scale (GCS) score 3–5, hemorrhage-induced midline shift or sustained deep coma) (note: ICH patients often have secondary epilepsy and, given that the decline in consciousness observed after secondary epilepsy is not positively correlated with the severity of intracranial hemorrhage, these patients cannot be assessed only by consciousness)
5. The presence of dementia (previously diagnosed by a medical institution) or significant post-stroke disability (modified Rankin Scale (mRS) ≥ 3 points [32])
6. A coagulation disorder caused by drugs or hematological disease (coagulation disorder is defined as < 50 × 10^9^/L platelets or ≥ 1.8 International Normalized Ratio (INR))
7. Allergy to opioids
8. Interfering test result, assessment or follow-up comorbidity (such as malignant tumor and respiratory diseases)
9. The presence of sinus arrest, borderline rhythm, grade II or higher atrioventricular block or malignant arrhythmia (individuals with bradycardia and arrhythmia who have a good pulse regulated by a pacemaker will not be excluded)
10. Is pregnant or lactating
11. Is currently participating in another drug study or clinical trial
12. The subject or guardian is unwilling to provide their ICF, or the subject is likely to persist with the study and follow-up; and
13. Subject participation in the study will increase their study-related risk and other reasons that make the subject unsuitable for the study as determined by the investigator

In particular, in this study, subjects with surgical emergency indications, such as neurosurgical drainage and decompression of the bone flap, will not be excluded.

### Randomization

Patients were randomized into two antihypertensive treatment groups, a sufficient analgesia combined with minimal sedation group (experimental group) or an antihypertensive drug treatment group (control group), by central randomization within 2 h of selection into the study. Randomized central control factors that need to be considered for centralized randomization include: (1) Emergency hematoma removal or neurosurgical intervention expected within 24 h (yes, no); (2) Use of antiplatelet or anticoagulant drugs within the past week (yes, no); and (3) Mechanical ventilation in a patient at the time of enrollment (yes, no). In this study, randomization will be performed using a randomization system in the Department of Biostatistics at Southern Medical University, Guangzhou, China. Each center will be assigned an account number and password to log in and enter the subjects’ basic information, control factors, etc., so that randomization can be completed by the system. The primary objective of this study was to explore the optimal antihypertensive regimen to use in ICH and to guide its clinical application. The control factors in this study were potential risk fachor and possible treatment for ICH, in order to explore safety and efficacy of most ICH subjects, they will not be excluded.

### Blinding

This study uses a single-blinded design in which the intervention is known to the investigators and executors but not to the subjects. All drugs used in this study will be administered only by intravenous (IV) infusion. The drug solutions will be prepared by the investigators after patient enrollment, labeled with the subjects’ code numbers and names (excluding grouping and drug information), and administered by IV infusion. Only the initial treatment and not the grouping and drug will be known to the subjects.

### Treatments

**Fig. 1:**
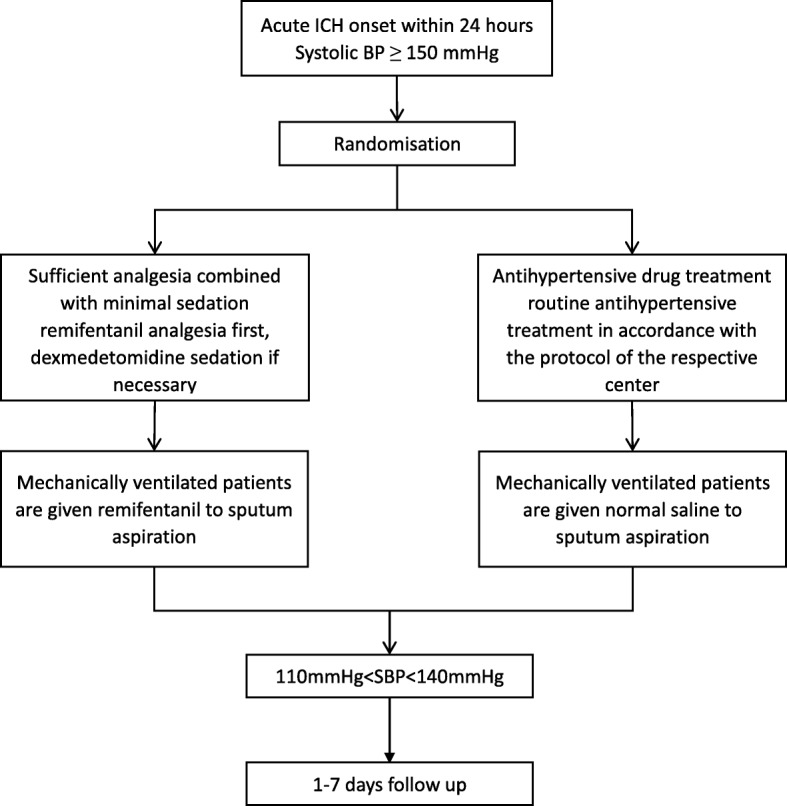
Flow diagram of the study

Figure 1 outlines the study protocol. A key principle is the rapid initiation of the randomized treatment. Investigators should aim to achieve a “door-to-needle” time of 2 h, which allows time to confirm eligibility, obtain informed consent, record several key clinical baseline parameters and identify the randomized assignment. The overall aim of antihypertensive treatment is to reduce systolic BP to 110–140 mmHg within 1 h of treatment initiation and maintain this systolic BP level for 7 days or until the subject is discharged from the ICU (in subjects who are transferred out of ICU in 7 days). In subjects who cannot be transferred out of the ICU after more than 7 days, the regimen will be administered for only the first 7 days, and other antihypertensive treatments may be given after 7 days, at which time the goal of antihypertensive therapy and the frequency of measurements will no longer be limited. BP will be measured every 10 min in the first 1 h after the start of treatment and then measured and recorded every 1 h. From the second day, it will be measured and recorded four times daily until the end of intervention.

In patients allocated to the experimental group, the overall treatment principle is to apply sufficient analgesia and sedation if necessary: remifentanil will be administered by IV infusion and maintained at a dose of 0.025 μg/kg/min in non-mechanically ventilated patients and a dose of 0.05 μg/kg/min in mechanically ventilated patients. BP will be measured after 10 min of continuous infusion; if systolic BP is still ≥ 140 mmHg, then dexmedetomidine will be applied using an infusion pump at a dose of 0.2 μg/kg/h. BP will be measured again after 15 min of continuous infusion of dexmedetomidine. If systolic BP is still ≥ 140 mmHg, the maintenance dose of dexmedetomidine will be increased 0.1 μg/kg/h. BP will be measured every 10 min during the infusion, and the maintenance dose of dexmedetomidine will be increased accordingly up to a maximum of 0.6 μg/kg/h. If BP is not reduced by the concurrent use of dexmedetomidine at its maximum dose, then the routine antihypertensive treatment of the respective center will be applied, and infusion antihypertensive treatment is recommended to rapidly reduce systolic BP to its target range. Mechanically ventilated patients will be given a rapid remifentanil (0.5 μg/kg) infusion as an analgesic prior to sputum aspiration to reduce procedure-related pain.

For patients allocated to the control group, routine antihypertensive treatment will be performed in accordance with the protocol of each respective research center. Urapidil, nicardipine, and labetalol will be used in this group. Urapidil will be used as follows: a slow IV injection of 10–15 mg and then IV pumping for maintenance at an initial rate of 2 mg/min, adjusted according to BP to a maximum of 9 mg/min. Nicardipine will be used as follows: IV pumping at 0.5 μg/kg/min adjusted according to BP to a maximum of 6 μg/kg/min. Labetalol will be used as follows: IV infusion for maintenance at 1–4 mg/min until the aim is reached. Endotracheal intubation disrupts the normal mucociliary movement of the respiratory tract, resulting in the retention of sputum, which increases the risk of ventilator-associated pneumonia. Coughing and effective sputum removal are important measures that prevent its occurrence in critically ill patients [33]. However, in patients with ICH, elevated BP and increased intracranial pressure caused by coughing can increase the risk of recurrent hemorrhage. No previous study has explored whether ICH patients who retain the ability to cough up sputum during aspiration have a better prognosis. Therefore, the mechanically ventilated patients in the control group will be administered a rapid physiological saline infusion as a controlled pretreatment.

### Outcomes

**Fig. 2:**
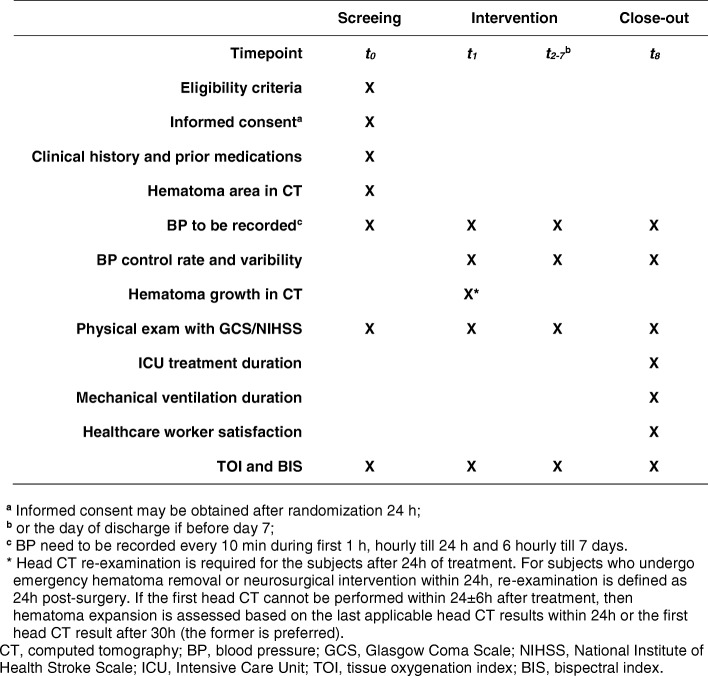
Schedule of assessments/data collection

Figure 2 outlines the schedule of assessments. All outcome measures should be measured according to a standard protocol, and BP will be recorded while the patient is supine, ideally using the same automated device throughout the trial.

#### Primary outcome

The primary outcome measures will be the systolic BP control rate at 1 h post treatment initiation. The number of patients in whom systolic BP decreased to < 140 mmHg at 1 h post treatment initiation will be compared to the total number in each group of patients.

#### Secondary outcomes

1. Hematoma growth at 24 h: early hematoma expansion is a major cause of neurological degeneration and poor clinical prognoses in ICH patients [34, 35]; most such cases occur within 6 h of ICH onset, and they rarely occur beyond 24 h. Head CT re-examination is required in the subjects after 24 h of treatment. Hematoma volume will be calculated using MIStar software (Apollo Medical Imaging Technology). For images in which hematoma volume cannot be calculated by the software, hematoma volume will be calculated by ABC/2 [36, 37]. Hematoma expansion is defined as V2 − V1 ≥ 12.5 cm^3^ or (V2 −V1)/V1>33% (V1 and V2 represent the hematoma volume in the two CT scans) [38]
2. BP variability: early BP variability is also a major factor that affects the prognosis of ICH [26]. Therefore, in this study, systolic BP, diastolic BP and mean arterial pressure were also recorded every hour from 2 to 24 h post treatment and monitored on days 2–7 of treatment every 6 h (four times per day). The BP coefficient of variation (CV) = (standard deviation of BP/mean of systolic BP)
3. Neurological function: remifentanil and dexmedetomidine have only a slight effect on consciousness and breathing and help patients with craniocerebral injury to stay conscious while under sedation. This allows real-time functional assessments of the nervous system [39–41]. Neurological function will be assessed once every morning using National Institutes of Health Stroke Scale (NIHSS) [42, 43] and GCS [44] scores, the Richmond Agitation-Sedation Scale (RASS) [45], the Nonverbal Adult Pain Assessment Scale (NVPS) [46], and the Reaction Level Scale (RLS) [47]
4. Duration of ICU treatment and mechanical ventilation: a previous randomized trial of patients with craniocerebral injury indicated that a remifentanil-based sedation strategy significantly reduced the amount of sedative used and shortened the time of mechanical ventilation [39]. The time during which ICU treatment and mechanical ventilation were required will be recorded when the patients leave the ICU
5. Healthcare worker satisfaction: because it uses precise dose control of analgesics and sedatives, this remifentanil-based sedation strategy could significantly reduce the frequency of assessments and dose adjustments required by healthcare workers, thus lowering their workload, increasing patient adherence, and improving healthcare worker satisfaction. A questionnaire measuring satisfaction has been designed based on the Copenhagen Psychosocial Questionnaire [48] to include a parameter for a self-assessed workload. When a patient leaves the ICU, each worker is required to fill in data according to their own feelings
6. Near-infrared spectroscopy (NIRS) is a non-invasive, non-ionizing method used for functional monitoring and imaging of brain hemodynamics and is used to study human brain function in both healthy states and a variety of pathologies [49, 50]. Therefore, we will continuously monitor the percutaneous cerebral tissue oxygenation index (TOI). We will also assess changes in the TOI after sputum aspiration in mechanical ventilation patients. Bispectral index (BIS) monitoring is one of several technologies used to monitor the depth of anesthesia. Most studies indicate that nociceptive stimulation causes a significant increase in the BIS, revealing the importance of controlling certain confounding variables such as the level of sedation [51]. BIS monitoring is used to continuously monitor consciousness levels

### Data and safety monitoring

Original case report forms (CRFs) will be archived and stored in an order corresponding to the subject codes after the completion of data entry and review. An independent Data and Safety Monitoring Board (DSMB) consisting of clinicians and biostatisticians will monitor the safety and progress of the trial. The DSMB will review all unblinded data, including those obtained in the treatment group, in addition to dropout and event rates at the end of the study. All data entered into the database will be locked after they have been reviewed, assessed, and confirmed to be correct, and no changes will be allowed to any locked data files. The DSMB will review all serious adverse events (SAEs) after 33% and 67% of the patients are enrolled, and the committee may modify or stop the trial at any point.

### Sample size

The BP control rate within 1 h of treatment initiation is the primary efficacy measure (qualitative data) in this trial. INTERACT 2, completed in 2013, showed that within 1 h of receiving intensive antihypertensive treatment, the BP control rate was 33.4% in patients [20]. Analgesia and sedation help to rapidly lower BP and reduce BP fluctuations in ICH patients. However, authoritative studies on the correlation between antihypertensive treatment in ICH and post-brain injury stress levels, cerebral oxygen metabolism and ICP levels are currently lacking. Based on past clinical experiences, the 1 h BP control rate is expected to be 34% among ICH patients who receive antihypertensive drugs and 51% among those who receive analgesia-first sedation therapy. Two-tailed tests with a significance level of 0.05 and a test power of 80%, as well as a parallel design, will be used in this trial. The sample size was estimated to be 132 subjects per group using the nQuery Advisor + nTerim 4.0 with a < 20% dropout rate. The final confirmed sample size is 165 subjects per group and 330 subjects in total.

### Statistical analysis

The intention-to-treat principle will be applied to the analysis. All analyses will be conducted with patients allocated to the group to which they were assigned at randomization. Baseline and demographic characteristics will also be summarized according to treatment group. Descriptive statistics: quantitative data will be expressed as the mean, standard deviation, and confidence interval. The minimum value, maximum value, P25, median, and P75 may also be shown when necessary. Non-parametric test results will be expressed as the median and mean rank. Ordinal data will be expressed as frequency distributions and corresponding percentages as well as median and mean rank. Qualitative data will be expressed as a positive rate, the number of positives and a denominator. All statistical analyses will be conducted using SAS 9.4 statistical software. All statistical inferences will be tested using two-tailed tests with a significance level of 0.05 and a confidence level of 95%.

The systolic BP control rate at 1 h post treatment initiation is the primary measure for efficacy assessment. Given that this measurement represents count data, the data will be compared using Pearson’s *χ*^2^ test. Logistic regression analysis will be used when other confounding factors are considered. Secondary efficacy measures: qualitative variables will be analyzed by Pearson’s *χ*^2^ test, ordinal variables will be analyzed by the Kruskal-Wallis test, and quantitative variables will be analyzed by one-way analysis of variance (ANOVA) (multiple groups), two-sample *t* test (two groups), or corresponding non-parametric tests. For variables that may affect outcomes, subgroup analyses may be performed by comparing the primary and secondary efficacy measures between the subgroups. Given our research purpose, the correlation coefficients for BP variability and hematoma growth rates will not be calculated directly. If the collinearity of the two is too large, some control methods will be adopted.

The adverse event (AE) incidence rate will be compared between the two groups using Pearson’s *χ*^2^ test, and all AEs that occur during the trial will be listed and described. Intra-group and inter-group comparisons of quantitative measures will be performed using the corresponding tests. Normal/abnormal changes in laboratory test results obtained before and after the study will be analyzed, and the relationships between abnormal changes and the investigational drug will also be examined.

### Pharmacovigilance plan

In this study, any new medically diagnosed disease, exacerbation of pre-existing comorbidity or other unpredictable medical events, aside from expected natural disease exacerbation caused by ICH progression (such as organ failure or other complications), that is identified in the subjects by the investigators after an intervention is applied in the clinical trial should be considered an AE. Serious adverse events (SAEs) refer to any adverse medical events observed, at any dose, that meet one or a multiple of the following criteria: death-causing, life-threatening, permanent or obvious disability/function impairment and important medical events. The severity of all AEs and their causal relationship with this study will be determined by the investigators. The safety of the subject should always be prioritized in a clinical study under any circumstance. Therefore, investigators should always be alert and try their best to monitor all potential AEs. Once an SAE occurs, it must be recorded in the CRF and reported to, and handled by, the corresponding emergency unit.

### Data management

Data collection and management in this study will be performed using the Electronic Data Capture (EDC) system. The system allows for web-based data entry and management. The data will be captured and entered at each participating hospital site using an electronic signature (unique user and password). All changes made following the electronic signing will have an electronic audit trail with a signature and date. Regional Coordinating Center (RCC) staff have access to online reports related to overall study status, the subject visit calendar, the CRF completion status and SAE reports so that they can assist with monitoring the quality of the data.

## Discussion

ICH remains a major public health problem. Several large-scale RCTs have indicated that while early antihypertensive treatment is recommended by guidelines, significant differences among large clinical studies conducted in recent years have provoked a great deal of controversy. The primary objectives of this study are to demonstrate that applying sufficient analgesia in combination with a minimal sedation program can rapidly and stably reduce increased BP in the early stage of ICH to thereby provide a theoretical basis for ICH emergency treatment strategies, improve short-term prognoses, shorten mechanical ventilation and ICU stays, reduce medical and social economic burdens, reduce the frequency of medical staff requirements to adjust the dose of antihypertensive drugs, reduce staff workload, and improve work efficiency. However, with regard for the patients, the regimen will fully protect the rights of the subjects, avoid pain, minimize the impact of the drugs on the mind and help with the timely observation of conditions. Hence, this approach is worthy of clinical application in developing countries. Compared with previous studies that focused only on elevated BP, our study is novel in that we explore an optimal treatment regimen for acute BP elevation developed based on pathophysiological changes and examinations of the in-depth significance of analgesic and anti-stress drugs during the acute phase of ICH. We believe that the exploration of solutions based on pathophysiological changes will provide new insights into treatments for acute-phase ICH. One of the limitations of this study is that the study and data collection can only be conducted in a critical care unit due to the side effects of analgesics and sedatives on breathing and consciousness. Therefore, the feasibility of our regimen in the general ward will need to be further explored. The enrollment of patients into this study began in June 2018. The results are expected in 2019 (Additional file 1).

### Trial status

Patient recruitment began in June 2018. At the time of manuscript submission, five patients had been recruited, and patient recruitment is expected to be completed in 1 year.

## Additional file

Additional file 1: SPIRIT checklist. (DOC 187 kb)

## Abbreviations

AE: Adverse event
APACHE II: Acute Physiology and Chronic Health Evaluation II
BIS: Bispectral index
BP: Blood pressure
CRF: Case report form
CT: Computed tomography
CV: Coefficient of variation
DSMB: Data and Safety Monitoring Board
EDC: Electronic Data Capture
GCS: Glasgow Coma Scale
ICF: Informed consent form
ICH: Intracerebral hemorrhage
ICP: Intracranial pressure
ICU: Intensive care unit
IEC: Institutional Ethics Committee
INR: International Normalized Ratio
mRS: Modified Rankin Scale
NIHSS: National Institutes of Health Stroke Scale
NIRS: Near-infrared spectroscopy
NVPS: Nonverbal Adult Pain Assessment Scale
RASS: Richmond Agitation-Sedation Scale
RCC: Regional Coordinating Center
RCT: Randomized controlled trial
RLS: Reaction Level Scale
SAE: Serious adverse event
TOI: Tissue oxygenation index
