# Supplementary material for: Safety and efficacy of applying sufficient analgesia combined with a minimal sedation program as an early antihypertensive treatment for spontaneous intracerebral hemorrhage: a randomized controlled trial
Source: Trials. 2018 Nov 6;19:607. doi: 10.1186/s13063-018-2943-6 (PMC6219080; doi:10.1186/s13063-018-2943-6)
Supplement: Supplementary file 1 — SPIRIT checklist. (DOC 187 kb) [file 13063_2018_2943_MOESM1_ESM.doc]

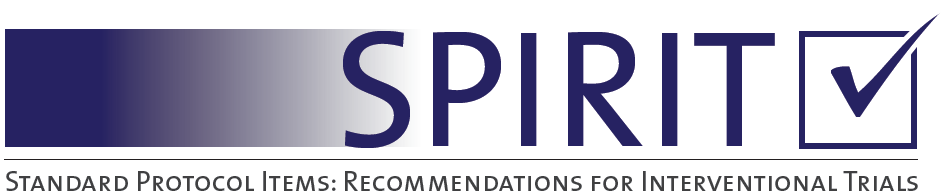


SPIRIT 2013 Checklist: Recommended items to address in a clinical trial protocol and related documents*

| Section/item | Item  No | Description | Addressed on page number |
| --- | --- | --- | --- |
| **Administrative information** | | |  |
| Title | 1✔ | Descriptive title identifying the study design, population, interventions, and, if applicable, trial acronym  Safety and Efficacy of Applying Sufficient Analgesia Combined with a Minimal Sedation Program as an Early Antihypertensive Treatment for Spontaneous Intracerebral Hemorrhage: A Randomized Controlled Trial. Showed on Page1, Line 1-4. | 1 |
| Trial registration | 2a✔ | Trial identifier and registry name. If not yet registered, name of intended registry  https://clinicaltrials.gov/ct2/show/NCT03207100  ClinicalTrials.gov ID is NCT03207100. Showed on Page 3, Line 50-51. | 3 |
|  | 2b | All items from the World Health Organization Trial Registration Data Set  N/A, no explanation in this article. | None |
| Protocol version | 3✔ | Date and version identifier  Version 4.0, Date 11/15/2017, but no explanation in this article. | None |
| Funding | 4✔ | Sources and types of financial, material, and other support  Current study required no funding. Showed on Page 23, Line 499-500. | 23 |
| Roles and responsibilities | 5a✔ | Names, affiliations, and roles of protocol contributors  Rui Dong, Department of Intensive Care Unit, The Third Affiliated Hospital of Southern Medical University, responsible for writing manuscripts.  Fen Li, Department of Intensive Care Unit, The Third Affiliated Hospital of Southern Medical University, responsible for writing manuscripts.  Ying Xu, Department of Biostatistics, School of Public Health, Southern Medical University, responsible for data analysis and randomisation.  Pingyan Chen, Department of Biostatistics, School of Public Health, Southern Medical University, responsible for data analysis and randomisation.  Marc Maegele, Institute for Research in Operative Medicine (IFOM), Witten/Herdecke University (Campus Cologne-Merheim), responsible for manuscript review.  Hong Yang, Department of Intensive Care Unit, The Third Affiliated Hospital of Southern Medical University, responsible for this study.  Wenjin Chen, Department of Neurosurgery, Xuanwu Hospital, Capital Medical University, responsible for this study.  All above are showed in Page 1, Line6-16 and Page 23,Line 502-50. | 1, 23 |
|  | 5b✔ | Name and contact information for the trial sponsor  Hong Yang: yhicu_1103@163.com  Wenjin Chen: [drchenwenjin@qq.com](mailto:drchenwenjin@qq.com)  All above are showed in Page 1, Line22-Page 2,Line 2. | 1, 2 |
|  | 5c✔ | Role of study sponsor and funders, if any, in study design; collection, management, analysis, and interpretation of data; writing of the report; and the decision to submit the report for publication, including whether they will have ultimate authority over any of these activities  Hong Yang and Wenjin Chen involved to for study design, collection, management, interpretation of data, interpretation of data, they have ultimate authority over any of these activities.  The article did not show all of these. Page 22, Line 505-506 explain part of these. | 22 |
|  | 5d✔ | Composition, roles, and responsibilities of the coordinating centre, steering committee, endpoint adjudication committee, data management team, and other individuals or groups overseeing the trial, if applicable (see Item 21a for data monitoring committee)  Explained in Page 17, Line 355-359. | 17 |
| Introduction |  |  |  |
| Background and rationale | 6a✔ | Description of research question and justification for undertaking the trial, including summary of relevant studies (published and unpublished) examining benefits and harms for each intervention  Research question were discussed in Page 3, Line 57-Page 5, Line 110 | 3-5 |
|  | 6b✔ | Explanation for choice of comparators  Explained in the Background, we used the results of previous studies as a control, showed in Page 4, Line 83-Page 5, Line 93. | 4-5 |
| Objectives | 7✔ | Specific objectives or hypotheses:  Objectives explained in paper, Page 7, Line 153-Page 8, Line 161.  Hypotheses explained in paper, Page 5, Line 110-Page 6, Line 115. | 5-6, 7-8 |
| Trial design | 8✔ | Description of trial design including type of trial (eg, parallel group, crossover, factorial, single group), allocation ratio, and framework (eg, superiority, equivalence, noninferiority, exploratory).  Explained in Methods/design, Page 6, Line 118-127 | 6 |
| Methods: Participants, interventions, and outcomes | | |  |
| Study setting | 9✔ | Description of study settings (eg, community clinic, academic hospital) and list of countries where data will be collected. Reference to where list of study sites can be obtained  Explained in Methods/design, Page 6, Line 130-132, and Table 1. | 6 |
| Eligibility criteria | 10✔ | Inclusion and exclusion criteria for participants. If applicable, eligibility criteria for study centres and individuals who will perform the interventions (eg, surgeons, psychotherapists)  Explained in Methods/design, Page 8, Line 163-Page 10, Line 210. | 8-10 |
|  | 11a✔ | Interventions for each group with sufficient detail to allow replication, including how and when they will be administered  Explained in Methods/design, Page 11, Line 239-Page 14, Line 290, and Figure 1. | 11-14 |
|  | 11b✔ | Criteria for discontinuing or modifying allocated interventions for a given trial participant (eg, drug dose change in response to harms, participant request, or improving/worsening disease)  For those with adverse reactions, we have corresponding dosage reduction measures, but this study does not differentiate between the subjects, the same group of subjects were taken the same intervention (same intervention drugs and dose strategy). So, no explanation in this article. | None |
|  | 11c✔ | Strategies to improve adherence to intervention protocols, and any procedures for monitoring adherence (eg, drug tablet return, laboratory tests)  The drug intervention in this study is mainly performed by intravenous infusion of medical staff, and the expected compliance is high. So, no explanation in this article. | None |
|  | 11d✔ | Relevant concomitant care and interventions that are permitted or prohibited during the trial  No explanation in this article. | None |
| Outcomes | 12  ✔ | Primary, secondary, and other outcomes, including the specific measurement variable (eg, systolic blood pressure), analysis metric (eg, change from baseline, final value, time to event), method of aggregation (eg, median, proportion), and time point for each outcome. Explanation of the clinical relevance of chosen efficacy and harm outcomes is strongly recommended.  Primary outcome explained in Page 14, Line 301-305.  Secondary outcomes explained in Page 14, Line 307-Page 16, Line 351.  All explained in Table 2. | 14-16 |
| Participant timeline | 13✔ | Time schedule of enrolment, interventions (including any run-ins and washouts), assessments, and visits for participants. A schematic diagram is highly recommended (see Figure)  Explained in Figure 1, and Table 2. | None |
| Sample size | 14✔ | Estimated number of participants needed to achieve study objectives and how it was determined, including clinical and statistical assumptions supporting any sample size calculations  Explained in Page 17, Line 365-Page 18, Line 379. | 17-18 |
| Recruitment | 15✔ | Strategies for achieving adequate participant enrolment to reach target sample size  Since our clinical trial is a multicenter study, each center will be need to include at least 24 cases based on sample size. The 15 centers in this study are located in 14 provinces in mainland China. Each center receives about 50-200 ICH patients every year, which can guarantee the completion of this study. Subject recruitment explained in Page 6, Line 132-Page 7, Line137. | 6, 7 |
| **Methods:** **Assignment of interventions (for controlled trials)** | | |  |
| Allocation: |  |  |  |
| Sequence generation | 16a✔ | Method of generating the allocation sequence (eg, computer-generated random numbers), and list of any factors for stratification. To reduce predictability of a random sequence, details of any planned restriction (eg, blocking) should be provided in a separate document that is unavailable to those who enrol participants or assign interventions  Explained in Page 10, Line 215-Line 217. | 10 |
| Allocation concealment mechanism | 16b✔ | Mechanism of implementing the allocation sequence (eg, central telephone; sequentially numbered, opaque, sealed envelopes), describing any steps to conceal the sequence until interventions are assigned  Explained in Page 10, Line 220-Page 11, Line 224. | 10, 11 |
| Implementation | 16c✔ | Who will generate the allocation sequence, who will enrol participants, and who will assign participants to interventions  There is no specific definition in this study. The trained participants can perform the above. So, no explanation in this article. | None |
| Blinding (masking) | 17a✔ | Who will be blinded after assignment to interventions (eg, trial participants, care providers, outcome assessors, data analysts), and how  Explained in Page 11, Line 230-237. | 11 |
|  | 17b✔ | If blinded, circumstances under which unblinding is permissible, and procedure for revealing a participant’s allocated intervention during the trial.  No unblinding is permissed. | None |
| **Methods: Data collection, management, and analysis** | | |  |
| Data collection methods | 18a✔ | Plans for assessment and collection of outcome, baseline, and other trial data, including any related processes to promote data quality (eg, duplicate measurements, training of assessors) and a description of study instruments (eg, questionnaires, laboratory tests) along with their reliability and validity, if known. Reference to where data collection forms can be found, if not in the protocol.  Explained in Page 20, Line 437-439. | 20 |
|  | 18b | Plans to promote participant retention and complete follow-up, including list of any outcome data to be collected for participants who discontinue or deviate from intervention protocols  This study only observed treated for 7 days or turned out of ICU, the follow-up time was short and the follow-up rate could be guaranteed. So, no explanation in this article. | None |
| Data management | 19✔ | Plans for data entry, coding, security, and storage, including any related processes to promote data quality (eg, double data entry; range checks for data values). Reference to where details of data management procedures can be found, if not in the protocol  Explained in Page 20, Line 432-436. | 20 |
| Statistical methods | 20a✔ | Statistical methods for analysing primary and secondary outcomes. Reference to where other details of the statistical analysis plan can be found, if not in the protocol  Explained in Page 18, Line 381-Page 19, Line 413. | 18, 19 |
|  | 20b✔ | Methods for any additional analyses (eg, subgroup and adjusted analyses)  There is no subgroup analysis in this study. | None |
|  | 20c✔ | Definition of analysis population relating to protocol non-adherence (eg, as randomised analysis), and any statistical methods to handle missing data (eg, multiple imputation)  Details are stated in the statistical analysis plan. So, no explanation in this article. | None |
| **Methods: Monitoring** | | |  |
| Data monitoring | 21a✔ | Composition of data monitoring committee (DMC); summary of its role and reporting structure; statement of whether it is independent from the sponsor and competing interests; and reference to where further details about its charter can be found, if not in the protocol. Alternatively, an explanation of why a DMC is not needed  Explained in Page 17, Line 355-359. | 17 |
|  | 21b✔ | Description of any interim analyses and stopping guidelines, including who will have access to these interim results and make the final decision to terminate the trial  Data monitoring committee can stop this guideline. Explain in Page 17, Line 361-363. | 17 |
| Harms | 22✔ | Plans for collecting, assessing, reporting, and managing solicited and spontaneously reported adverse events and other unintended effects of trial interventions or trial conduct  Explained in Page 19, Line 415-Page 20, Line 429. | 19-20 |
| Auditing | 23 | Frequency and procedures for auditing trial conduct, if any, and whether the process will be independent from investigators and the sponsor.  This process will be independent from investigators and the sponsor. So, no explanation in this article. | None |
| Ethics and dissemination | | |  |
| Research ethics approval | 24✔ | Plans for seeking research ethics committee/institutional review board (REC/IRB) approval  The institutional ethics committee of The Third Affiliated Hospital of Southern Medical University, reference number 201711009. Explained in Page 6, Line 124-127. | 6 |
| Protocol amendments | 25✔ | Plans for communicating important protocol modifications (eg, changes to eligibility criteria, outcomes, analyses) to relevant parties (eg, investigators, REC/IRBs, trial participants, trial registries, journals, regulators)  No explanation in this article | None |
| Consent or assent | 26a✔ | Who will obtain informed consent or assent from potential trial participants or authorised surrogates, and how (see Item 32)  Explained in Page 7, Line 143-146. | 7 |
|  | 26b✔ | Additional consent provisions for collection and use of participant data and biological specimens in ancillary studies, if applicable  None additional data and biological specimens in this study. | None |
| Confidentiality | 27✔ | How personal information about potential and enrolled participants will be collected, shared, and maintained in order to protect confidentiality before, during, and after the trial.  Explain in Page 17, Line 359-361. | 17 |
| Declaration of interests | 28✔ | Financial and other competing interests for principal investigators for the overall trial and each study site  None declared | None |
| Access to data | 29✔ | Statement of who will have access to the final trial dataset, and disclosure of contractual agreements that limit such access for investigators  Explained in Page 17, Line 359-361. | 17 |
| Ancillary and post-trial care | 30✔ | Provisions, if any, for ancillary and post-trial care, and for compensation to those who suffer harm from trial participation  No explanation in this article | None |
| Dissemination policy | 31a✔ | Plans for investigators and sponsor to communicate trial results to participants, healthcare professionals, the public, and other relevant groups (eg, via publication, reporting in results databases, or other data sharing arrangements), including any publication restrictions  None plan. | None |
|  | 31b✔ | Authorship eligibility guidelines and any intended use of professional writers  None. | None |
|  | 31c | Plans, if any, for granting public access to the full protocol, participant-level dataset, and statistical code.  None plan. | None |
| Appendices |  |  |  |
| Informed consent materials | 32✔ | Model consent form and other related documentation given to participants and authorised surrogates.  None. | None |
| Biological specimens | 33 | Plans for collection, laboratory evaluation, and storage of biological specimens for genetic or molecular analysis in the current trial and for future use in ancillary studies, if applicable  None. | None |

*It is strongly recommended that this checklist be read in conjunction with the SPIRIT 2013 Explanation & Elaboration for important clarification on the items. Amendments to the protocol should be tracked and dated. The SPIRIT checklist is copyrighted by the SPIRIT Group under the Creative Commons “[Attribution-NonCommercial-NoDerivs 3.0 Unported](http://www.creativecommons.org/licenses/by-nc-nd/3.0/)” license.
